# When Does Educational Level Diversity Foster Team Creativity? Exploring the Moderating Roles of Task and Personnel Variability

**DOI:** 10.3389/fpsyg.2021.585849

**Published:** 2021-03-05

**Authors:** Weixiao Guo, Chenjing Gan, Duanxu Wang

**Affiliations:** ^1^Business School, Ningbo University, Ningbo, China; ^2^School of Management, Zhejiang University, Hangzhou, China

**Keywords:** team creativity, educational level diversity, task variability, personnel changes, moderating analysis

## Abstract

This study explores how the variability of the work environment shapes the impact of educational level diversity on team creativity. By adopting an integrative framework—“status characteristics–information elaboration” model as a theoretical lens, we propose and examine the moderating roles of task and personnel variability in educational level diversity–team creativity relationship. Utilizing multiple survey data collected from 90 knowledge work teams, the empirical results indicate that educational level diversity is more conducive to team creativity when teams are confronted with more variable tasks and when teams experience less frequent personnel changes. The findings of this study provide valuable insight on the conditions under which team diversity’s information potential is more likely to realize and contribute to a more context-based understanding of the relationship between diversity and creativity.

## Introduction

Confronted with turbulent circumstances, organizations become increasingly dependent on teams to carry out creative work to maintain the flexibility and sustained competitive advantages (Horwitz and Horwitz, 2007; Kearney et al., 2009; Hoever et al., 2012; Van Dijk et al., 2012; Van Veelen and Ufkes, 2019). Despite the recognition of the importance of team creativity, which is defined as the generation of novel and useful ideas regarding products, processes, and procedures (Amabile, 1996; Shin and Zhou, 2007), the conditions that foster team creativity require further investigation (Shalley et al., 2004; Shin and Zhou, 2007; Hoever et al., 2012). One of the most concerned and contentious issues is the creative impact of team demographic diversity (e.g., sex, educational level) (i.e., Milliken and Martins, 1996; Joshi, 2006; Bell et al., 2011; Guillaume et al., 2017).

In the past few decades, research on team demographic diversity–creativity relationship has not arrived at a consistent result. Some argue that demographic diversity may bring the risk of interpersonal conflicts, undermine team coordination, and hinder team creativity from the perspective of social categorization (Williams and O’Reilly, 1998; Dahlin et al., 2005; Van der Vegt et al., 2006; Joshi et al., 2011), whereas others argue that demographic diversity is thought to foster creativity by providing heterogeneous knowledge, experience, and perspectives from the perspective of information elaboration (Milliken and Martins, 1996; Nijstad and Paulus, 2003; Hülsheger et al., 2009; Bell et al., 2011; Curşeu et al., 2012; Van Dijk et al., 2012). Besides, from the perspective of status characteristics, demographic diversity is deemed to indicate the differences in possession of socially valued assets or resources, referring to that employees with higher educational level or longer tenure easier make their voices heard, and such status characteristic differences may lead to the suppression of some team members’ opinions and impede team creativity (Harrison and Klein, 2007; Bell et al., 2011).

As to reconcile the mixed results of the relationship between demographic diversity and team creativity, current research has moved away from investigating the main effect and shifted its focus on the context under which demographic diversity teams could realize their creative potential (i.e., Zhang, 2016; Guillaume et al., 2017; Lu et al., 2018). The present study builds on this trend and aims to identify the contingent factors between educational level diversity and team creativity by examining the moderating roles of task and personnel variability. We focus on educational level diversity primarily because it becomes one of the management challenges on how to make members with diversified educational levels play the synergy effect of teamwork under the contemporary trend where people have more discretion at education, such as junior college/vocational education and undergraduate and graduate (master or doctoral).

The present study extends the pieces of literature on diversity and creativity in several ways. First, in consistency with the latest conceptual framework proposed by Harrison and Klein (2007), this study takes the specific form of educational level diversity (separation and disparity) into consideration. By proposing an integrative theoretical framework—“status characteristics–information elaboration” model, this study offers a comprehensive rationale for understanding the relationship between educational level diversity and team creativity. Second, this study answers the call for context-based research in the field of diversity–creativity relationship by examining the moderating roles of task and personnel variability. Adopting the theoretical lens of the “status characteristics–information elaboration” model, this study is devoted to exploring the conditions under which the positive information synergy of educational level diversity would be realized, whereas the negative status-based problems would be avoided. Finally, by conducting multiple surveys in the field, the study addresses the current lack of empirical evidence on team creativity.

## Theory and Hypotheses Development

### A Closer Look at Team Educational Level Diversity

Team diversity is generally defined as the distributional differences among team members with respect to a specific personal attribute (Jackson et al., 1995; Harrison and Klein, 2007). As suggested by the recent advanced theoretical framework, team diversity that involves most demographic characteristics (e.g., sex, age, or educational level) can be displayed as three distinctive patterns—separation, variety, and disparity (Harrison and Klein, 2007). The three manifestations of team diversity seem to be equivalent when each is minimized; with increasing diversity, they become more differentiated in shape, meaning, relevance to key theoretical perspectives, and possible consequences.

Separation diversity indicates differences in position or opinion among team members (Harrison and Klein, 2007). When focusing on separation, team diversity indicates the extent to which disagreement or opposition among team members is present. Maximum separation occurs when team members are equally split and at opposing end-points along the continuum of a concerned attribute. Based primarily on social categorization theory, the literature tends to propose that team separation diversity has a negative impact on team identification, cohesion, and cooperation.

Variety diversity indicates differences in kind or category of knowledge or experience among team members (Harrison and Klein, 2007). When variety is stressed, team diversity indicates the extent to which the team knowledge base is redundant. Maximum variety occurs when each member represents a distinctive category of the concerned attribute. Based primarily on information elaboration theory, the literature tends to propose that team variety diversity has a positive impact on access to a wider range of knowledge and cognitive resources.

Disparity diversity indicates differences in possession of socially valued assets or resources such as pay and status among team members (Harrison and Klein, 2007). When focusing on disparity, team diversity indicates the extent to which team members’ viewpoints and opinions are treated unequally (Bell et al., 2011). The maximum disparity occurs when one team member dominates the others during task execution. Although disparity diversity has rarely been addressed in the field of team diversity, the differences in the proportion of valued resources such as pay and status among team members are likely to give rise to conformity, vicious competition, and information asymmetry (Harrison and Klein, 2007).

### Research on Educational Level Diversity and Team Creativity

Most of the extant research on the relationship between educational level diversity and team creativity, which adopted the perspective of social categorization and/or information elaboration, stressed the separation, and/or variety diversity (e.g., Van der Vegt and Bunderson, 2005; Somech, 2006; Shin and Zhou, 2007; Cannella et al., 2008). Specifically, on the one hand, differences in educational level are considered as the basis of team separation or subdivision and are proposed—from the perspective of social categorization—to provoke interpersonal conflicts and cooperation dilemmas, which are detrimental to team creativity (e.g., Milliken et al., 2003; Shin and Zhou, 2007). On the other hand, differences in educational level are considered as an indicator of variety and non-redundancy of cognitive resources and are proposed—from the perspective of information elaboration—to provide intellectual support and optimized information processing, which are beneficial to team creativity (e.g., Ancona and Caldwell, 1992; Shin and Zhou, 2007). Unfortunately, however, differences in educational level have seldom been viewed from the form of disparity, which refers to the vertical distributional differences of the possession of valued and desirable task-related resources among team members (Jackson et al., 1995; Harrison and Klein, 2007).

As suggested, the creative impact of educational level disparity diversity can be elaborated based on status characteristics theory (Bunderson, 2003; Harrison and Klein, 2007). The team status of a member is understood to depend on his/her performance expectations, which are based on his/her possessions and the extent to which these possessions are important for task completion and goal attainment (Berger et al., 1986; Bunderson, 2003). When an individual is expected to achieve higher performance, he/she will be given a more prominent role, and his/her opinions are likely to be widely acknowledged and deeply processed. Educational level is likely to influence performance expectations and team status (Berger et al., 1986; Bunderson, 2003; Van der Vegt et al., 2006). With increased educational level disparity diversity, the distribution of educational resources is more centralized, and the gap between members of “higher” and “lower” status is greater.

Based on status characteristics theory, it is assumed that educational level diversity, typically involving the unequal team status of members, negatively affects divergent thinking, in-depth communication, and knowledge use. On the one hand, members with higher team status are likely to shift their focus from accomplishing tasks to retaining their grip on influence and power (Klein et al., 2004), most likely by monopolizing critical task-related resources, dominating team processes, and imposing their viewpoints upon others. On the other hand, members with lower team status, whose perspectives are seldom taken into account, are likely to refrain from expressing divergent opinions and to submit to members with higher team status (Harrison and Klein, 2007).

In summary, the three connotations of team educational level diversity differ in their possible consequences consistent with the theoretical perspectives that are most relevant to them. Therefore, to further examine the complicated relationship between educational level diversity and team creativity, researchers should adopt a more integrative and comprehensive theoretical framework. Combining the trend that the research focus in the field of team diversity should be shifted to identifying critical contexts, under which team creativity is more likely to be facilitated or hindered, to provide greater theoretical and practical implications. The following section proposes and explicates hypotheses about the moderators intervening in the relationship between educational level diversity and team creativity.

### Moderators in the Creative Impact of Team Educational Level Diversity

**FIGURE 1:**
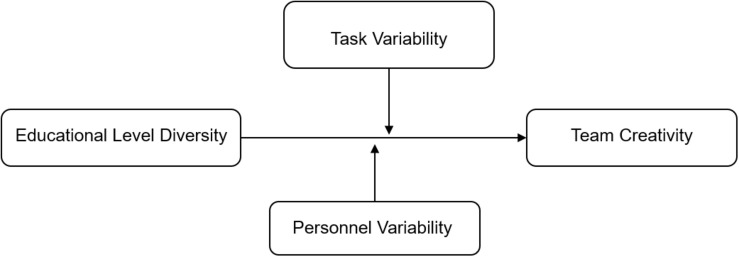
Research model of the moderating roles of task and personnel variability between educational level diversity and team creativity.

This study focuses on the separation and disparity form of educational level diversity. Although the existing literature has made great progress in understanding the moderators between diversity–creativity relationship (e.g., Shin and Zhou, 2007), few of them take the negative impact of diversity-related inequal status into consideration (Van Knippenberg and Schippers, 2007; Hoever et al., 2012). To address this gap, we propose the integrative “status characteristics–information elaboration” theoretical framework and hypothesize the moderating roles of task and personnel variability. Figure 1 depicts the theoretical model.

### Moderating Role of Task Variability

Task variability represents the extent to which a task is varied and variable (cf. Dewar et al., 1980; Diefendorff et al., 2006). Variable tasks are characterized as uncertain and complex and involve a much wider range of task-related knowledge, skills, and distinctive activities, whereas unvaried tasks are predictable, repetitive, and well defined, and they can be performed using standardized procedures (Diefendorff et al., 2006; Rico et al., 2008; Rousseau and Aube, 2010). In extant research, the extent to which team tasks are variable has been widely acknowledged as a significant impact factor for teamwork (Bowers et al., 2000; Diefendorff et al., 2006; Horwitz and Horwitz, 2007; Rico et al., 2008; Rousseau and Aube, 2010; Keller, 2012; Van Dijk et al., 2012).

The content and essence of a task determine the knowledge, skills, and capabilities required to perform it and are thought to interfere with the impact of educational level diversity on team creativity. Consistent with status characteristics theory, the difference among team members in the educational level is commonly regarded as a source of inequity of members’ influences on team collective decisions and actions. Such inequity affects the sense-making process of less-educated team members and is considered to suppress their different voices and fresh ideas, which undermines team creativity. Invariable tasks, for which limited and well-defined knowledge and skills are required, are repetitively executed over time, and team members tend to get stuck in the mindset and be restrained in their fixed roles.

Furthermore, teams in charge of tasks lacking variability tend to develop and comply with sets of standard processes and procedures (Diefendorff et al., 2006). Such routine tasks blind team members to the utilization of divergent cognitive resources because they prefer to rely on simple cues, stereotypes, and standard procedures rather than exploring and processing more task-related information (Kearney et al., 2009; Petty et al., 2009). The inequity of members’ influences on team collective decisions and actions will be strengthened during the repetitive execution of routine tasks. Therefore, when teams are confronted with unvaried tasks, the higher the level of team educational level diversity, the more team creativity is likely to suffer.

Conversely, when responding to variable tasks, teams tend to remain flexible instead of settling into a routine, which is likely to attach increasing significance to the potential of team educational level diversity for information elaboration. Teams need to develop a shared understanding of the new task and re-identify critical resources required for task completion. Accordingly, the expected performance contribution of each member in different tasks will be adjusted, which reduces the possibility for teams to form a fixed unequal social status and inequal treatment to members’ opinion. Thus, task variability would weaken the negative impact of educational level diversity on team creativity by hindering the emergence and/or solidification of social hierarchy/inequal status within the team. Furthermore, variable tasks can motivate team members to engage in cognitive activities that affect the extent to which task-related information is explored and processed (Kearney et al., 2009), the tolerance of ambiguity, and team creativity (Cacioppo et al., 1996). Because variable tasks involve many exceptions, unexpected situations, possibilities, and alternatives and require a larger knowledge base and more in-depth discussions, team members are encouraged to share unique information, propose different perspectives, make fresh attempts to perform the task, and seek novel solutions (Petty et al., 2009). Therefore, when teams are confronted with variable tasks, team educational level diversity is more likely to be regarded as a cognitive conduit and a large information repository for team creativity. The higher the level of team educational level diversity, the more likely it is that team creativity will be induced. Therefore, we propose the following hypothesis.

*Hypothesis H1*: The relationship between team educational level diversity and team creativity is moderated by team task variability such that team educational level diversity is more positively related to team creativity when there is a higher level of task variability.

### Moderating Role of Personnel Variability

Personnel change has become increasingly prevalent in managerial practice, aggravating the turbulence and fierceness of the competition. The extent to which personnel variability is present in teams is thought to provide crucial contextual influence on team creativity and calls for further understanding. In this study, we propose that team personnel variability moderates the relationship between team educational level diversity and team creativity. Specifically, in teams with a more frequent personnel change, team members are likely to attach great importance to the establishment and preservation of a relatively reliable and fixed mode at the expense of fresh attempts by trying to remain immune to the changes of team members (Madsen et al., 2003). This pattern seems to intensify the dominance of certain team members with higher team status while keeping members with lower team status from sharing their unique perspectives or proposing different opinions (Gruenfeld et al., 1996). Moreover, personnel variability is accompanied by changes in the quality and quantity of the team’s knowledge base. Because team educational level diversity indicates the distinctiveness of the task-related resources possessed by each member, from the perspective of information elaboration, it is believed that when there is more team educational level diversity, team knowledge is less redundant, and team creativity will be more improved.

By contrast, in teams with a less frequent personnel change, the accumulation of collaboration provides more opportunities for team members to develop mutual understanding other than using an educational level as the main evidence for performance expectations. Also, the lower level of personnel variability may give full play to the positive synergy brought by educational level diversity (Horwitz and Horwitz, 2007). It has been suggested that differences in demographic characteristics make it easier for teams to arrive at a consensus on the distribution of the team’s cognitive resources and to avoid cognitive redundancy and replicative efforts (Lewis et al., 2007; Wageman et al., 2012). Relevant research indicates that collective working experience enhances team identification, cohesiveness, trust, and the sense of belonging (Gruenfeld et al., 1996; Williams, 2001; Webber and Donahue, 2001; Van der Vegt et al., 2010), which are likely to undermine the negative impact of team educational level diversity. Based on the discussion earlier, we propose the following hypothesis.

*Hypothesis H2*: The relationship between team educational level diversity and team creativity is moderated by team personnel variability such that team educational level diversity is more positively related to team creativity when there is a lower level of personnel variability.

## Materials and Methods

### Sample and Data Collection

Data were collected from 90 teams in 36 organizations in China that were engaged in the industries of manufacturing, real estate, finance, information technology, software development, telecommunications, energy, and consulting. All these teams were in charge of knowledge-based tasks, such as product development, providing solutions, architecture design, and customer service. Initially, we contacted the immediate superiors of these teams or the middle-rank managers of the companies, briefly introduced the purpose of the survey, and promised the exclusive use of data for research and feedback in return. After obtaining their permission, we asked for a coordinator’s help in distributing and collecting questionnaires to ensure the efficiency of the process.

Of 122 invited teams, responses were received from 99 (81.1%). We filtered the data by omitting questionnaires with the same score for all items or more than half missing values and excluding teams that lacked data from team leaders and/or 50% or more of the members (see Rulke and Galaskiewicz, 2000; Bunderson, 2003). The final sample consisted of 373 valid individual cases from 90 teams (73.8%), including 17 R&D teams (18.9%), 25 marketing and sales teams (27.8%), 23 technical service teams (25.6%), and 25 teams with other functions (27.8%). Team sizes ranged from 3 to 13 members (mean = 6.18, *SD* = 2.80). The average team longevity is 39.7 months (*SD* = 30.3). The average age of the team members was 29.4 years (*SD* = 6.34), 60.5% of the team members were male, and 86.0% of the team members had earned a college diploma or above.

### Measures

All measures were adapted from well-established measures published in top academic journals according to our research. We created Chinese versions of these measures by strictly following a translation-back translation procedure. Additionally, data were collected from multiple sources to minimize potential common method biases. Specifically, team educational level diversity was calculated based on team members’ demographic data, team creativity was rated by team leaders, task variability was rated by team members, and team personnel variability was obtained from archival data.

#### Team Educational Level Diversity

Team educational level diversity was measured with the coefficient of variation indexes (standard deviation divided by the mean, Allison, 1978). Educational level was divided into five grades, from “1” for “high school or below” to “5” for “doctoral or above.” The mean coefficient of variation of educational level across the sample of teams was 0.20 (*SD* = 0.15).

#### Team Creativity

Team creativity was measured by six items according to a scale developed by Neil and Michael (1998) and Chen (2006). Sample items included “Our team always expands new knowledge and skills related to the task” and “Our team always proposes original solutions.” For each item, the leaders were asked to indicate the extent to which they agreed with the items on a five-point Likert scale ranging from 1 (absolutely disagree) to 5 (absolutely agree). The Cronbach’s alpha for the scale was 0.89.

#### Task Variability

Task variability was measured by four items adapted from a scale developed by Robert et al. (1980) and reverse scored. Sample items included “Members of our team do the same job in the same way every day” and “Most jobs of our team are almost the same.” For each item, team members were asked to indicate the extent to which they agreed with the items on a five-point Likert scale ranging from 1 (absolutely disagree) to 5 (absolutely agree). The Cronbach’s alpha for the scale at the individual level was 0.83.

#### Personnel Variability

Personnel variability was calculated based on archival data using the formula of dividing the number of personnel change incidents during the last year by the team size (Arrow and McGrath, 1995). We selected a 1 year period for this study under the assumption that 1 year was sufficient for team personnel change and for new members to have observable effects on team outcomes (see Van der Vegt et al., 2010).

#### Control Variables

We controlled several variables to enhance the validity of the results. Consistent with relevant research, we controlled team type, size, longevity (the average team tenure of team members), and sex (the percentage of women in teams). Also, we also controlled the mean of team members’ educational level to explore whether team educational level diversity explained team creativity after controlling for the impact of elevated levels of these continuous variables (Bell et al., 2011).

### Level of Analysis

We examined within-group agreement (rwg) values based on uniform null distribution before aggregating task variability from the members’ ratings to the team-level variable (James et al., 1984). The median of the rwg of task variability was 0.89, which was well above the conventionally acceptable rwg value of 0.70 (James et al., 1984). Additionally, we calculated the intraclass correlation coefficient ICC (1) and ICC (2). The means of ICC (1) and ICC (2) for task variability were 0.28 and 0.62, respectively. As shown, the indexes of ICC (1) were greater than 0.12 (James, 1982), and the indexes of ICC (2) were greater than 0.60 (Bliese, 2000). Accordingly, task variability was qualified for aggregation to the team level.

## Results

Table 1 presents the means, standard deviations, and correlations for the study variables. Consistent with most findings, team educational level diversity, and team creativity had a non-significant relationship (*r* = −0.09, *p* > 0.1).

**TABLE 1:** Means, standard deviations, and correlations (*n* = 90).

Variables	M	*SD*	1	2	3	4	5	6	7	8	9	10	11	12	13	14
1. R and D^a^	0.19	0.39														
2. Sales^a^	0.28	0.45	−0.30**													
3. Technical service^a^	0.26	0.44	−0.28**	−0.36***												
4. Other^a^	0.28	0.45	−0.30**	−0.39***	−0.36***											
5. Team size	6.18	2.80	−0.09	−0.01	0.12	−0.02										
6. Team longevity	39.69	30.33	−0.21*	−0.12	0.11	0.19*	0.24									
7. Gender^b^	0.37	0.33	−0.34**	0.29**	−0.17	0.17	0.08	−0.03								
8. Educational level	2.62	0.81	0.26*	−0.32**	0.17	−0.07	0.10	−0.07	−0.19*							
9. Age diversity	0.12	0.08	−0.30**	−0.02	−0.13	0.41***	0.04	0.32**	0.05	−0.34**						
10. Gender diversity	0.26	0.22	−0.16	0.04	−0.01	0.10	0.19	−0.02	0.30**	0.17	−0.02					
11. Tenure diversity	0.57	0.33	−0.26*	0.28*	0.02	−0.07	0.08	−0.00	0.17	0.09	0.19	0.06				
12. ELD	0.20	0.15	−0.14	0.04	−0.00	0.09	0.20*	0.16	0.02	−0.37***	0.43***	−0.11	0.20			
13. Team creativity	4.00	0.66	0.08	−0.01	−0.03	−0.04	−0.22*	−0.19*	−0.10	−0.01	−0.15	−0.04	−0.07	−0.09		
14. Task variability	1.50	0.58	0.23*	−0.15	0.15	−0.19*	0.07	−0.23*	−0.21*	0.48***	−0.23*	0.04	0.11	−0.13	0.05	
15. PV	0.94	0.91	−0.08	−0.10	0.33**	−0.15	−0.15	0.09	−0.06	0.08	−0.07	0.16	−0.13	−0.20	0.03	−0.12

^*a*^Dummy variable, R&D = Research and development team, Other teams include human resource, finance, production, etc. ^*b*^Dummy variable for gender, mean gender of each team is reported. ELD, educational level diversity; PV, personnel variability. *p < 0.05, **p < 0.01, ***p < 0.001.

We adopted hierarchical regression analyses to further examine the hypotheses. To minimize any potential threats of multicollinearity, we standardized predictor variables before calculating the cross-product terms (Aiken and West, 1991). We entered the control variables into Model 1 and added the independent variable and moderators into Model 2. The interaction terms were added into Model 3. The results are displayed in Table 2.

**TABLE 2:** Summary of hierarchical regression analysis results (*n* = 90).

Variables	Team creativity		
	Model 1	Model 2	Model 3
**Step 1: control variables**			
RandD^a^	−0.01	−0.02	−0.05
Technical service^a^	−0.01	−0.01	0.01
Other^a^	0.04	0.05	0.05
Team size	−0.17	−0.17	−0.24
Team longevity	−0.12	−0.12	−0.13
Gender	−0.11	−0.11	−0.18
Educational level	−0.07	−0.09	0.00
Age diversity	−0.14	−0.15	−0.16
Gender diversity	0.03	0.03	0.06
Tenure diversity	−0.02	−0.03	0.03
**Step 2: independent variable and moderators**			
Educational level diversity		0.01	0.16
Task variability		0.04	0.06
Personnel variability		0.01	−0.16
**Step 3: moderation**			
ELD × task variability			0.30*
ELD × personnel variability			−0.27*
*R*^2^	0.09	0.09	0.18
△*R*^2^	0.09	0.00	0.09
△*F*	0.79	0.37	4.18*

ELD, Educational level diversity, a dummy variable. Standardized regression coefficients are reported. *p < 0.05.

As shown in Model 2, no significant relation was found between team educational level diversity and team creativity. The moderating effects were examined in Model 3. As indicated, both team educational level diversity × task variability (β = 0.44, *P* < 0.05) and team educational level diversity × personnel variability (β = −0.30, *P* < 0.05) were significantly related to team creativity and explained a significant amount of variance (△*R*^2^ = 0.09, △*F* = 4.23, *p* < 0.05).

**FIGURE 2:**
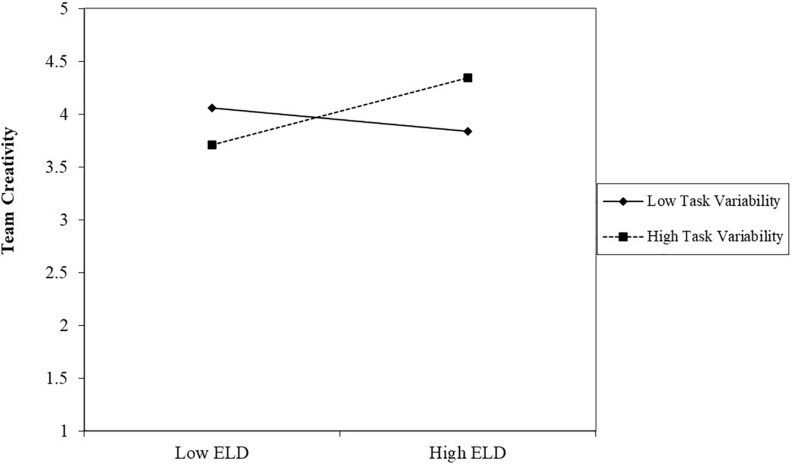
The moderating role of task variability between educational level diversity and team creativity.

**FIGURE 3:**
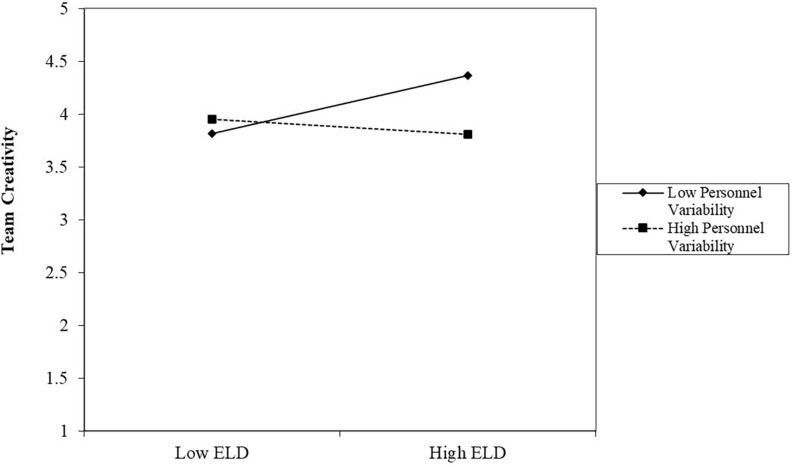
The moderating role of personnel variability between educational level diversity and team creativity.

Figures 2, 3 describe the patterns of the moderators’ impact on the relationship between team educational level diversity and team creativity. As depicted in Figure 2, team educational level diversity is more positively related to team creativity when task variability was higher. The simple slope test further showed that at a high level of task variability, educational level diversity was positively and significantly related to team creativity (β = 0.32, *p* < 0.01); however, at a low level of task variability, the relationship between educational level and team creativity was not significant (β = −0.11, *p* > 0.1). Thus, Hypothesis 1 was supported. As depicted in Figure 3, team educational level diversity was more positively related to team creativity when team personnel variability was lower. The simple slope test further showed that at a low level of personnel variability, educational level diversity was positively and significantly related to team creativity (β = 0.28, *p* < 0.05); however, at a high level of personnel variability, the relationship between educational level and team creativity was not significant (β = −0.07, *p* > 0.1). Thus, Hypothesis 2 was supported.

## Discussion

In recent decades, scholars have embraced the advantages and avoided the impediments of diversity in teams. Considering the prominence of team creativity and the emergent call for more context-based research, we focused on the contextual boundaries in which team educational level diversity was more likely to be conducive to team creativity. As hypothesized, both the variability of task and personnel moderated the relationship between team educational level diversity and team creativity. Specifically, the empirical results indicated that when teams were confronted with more variable tasks or experienced a lower frequent personnel change, team educational level diversity was more likely to facilitate team creativity.

### Theoretical and Practical Implications

Adopting a nuanced view, we paid particular attention to the conceptualization of team educational level diversity and advanced into the uncharted territory of team diversity research by taking into account the creative effect of team educational level disparity diversity. Although scholars have attached great significance to the potential of assembling members with different educational levels, the substance and pattern of these differences have seldom been examined. The most recent conceptual framework indicated that the ambiguity of the creative effect of team diversity was, to some extent, attributable to ignorance of the complexity of team diversity. In accordance with Harrison and Klein’s (2007), we stressed and addressed the lack of understanding of the disparity pattern in team diversity. Moreover, integrating the disparity pattern of team educational level diversity, we explained the rationale for how educational level diversity affects team creativity in the light of social categorization theory, information elaboration theory, and status characteristics theory, which helped us to gain a much more comprehensive understanding of the creative impact of team educational diversity.

This study also contributed to the team creativity literature. Because creativity is important at the team level for the survival and development of organizations, there is a need for deeper understanding and empirical evidence on how diversity affects team creativity (e.g., Shin and Zhou, 2007). We conducted a field study and collected data from multiple sources to achieve higher ecological validity, complementing the laboratory studies that are more frequently conducted in team creativity research; furthermore, we increased the reliability of the results by minimizing common method biases.

Finally, we answered the call for more context-based research in the field of diversity and creativity by theorizing and examining the moderating role of task and personnel variability (Jackson et al., 2003; Rico et al., 2008). The empirical results suggested that team educational level diversity may foster or impede team creativity, which was contingent on the extent to which tasks and personnel were changeable in teams. The coexistence of potential and threats in the differences of educational level among team members calls for greater research attention to clarify the substance of diversity and to investigate the boundary conditions that encourage or inhibit the expected consequences.

This study has certain practical implications. On the one hand, the possible consequences of differences in demographic characteristics should be considered more comprehensively when building a team. To address increasingly fierce competition, it is common to adopt teams consisting of members with different demographic characteristics. However, in addition to the benefits of diverse information, there may be threats induced by social categorization and the inequity of task-related resources largely ignored. Therefore, it is more important to identify the forms of the distributional difference in team members’ demographic characteristics, to estimate possible pros and cons, and to make real-time adjustments rather than focusing simply on superficial composition. On the other hand, the contextual condition should be considered when team diversity is adjusted. The results of this study indicate that less variable tasks and a higher team membership change tend to invoke a negative impact of team educational level diversity on team creativity, whereas variable tasks and a lower team membership change may have positive effects on team creativity.

### Limitations and Directions for Future Research

Firstly, the cross-sectional design of this research failed to provide direct evidence of a causal relationship. Although our hypotheses were theoretically driven, future research should adopt a longitudinal or experimental design to provide more convincing evidence of causality. Secondly, no objective measures are taken of team creativity. Although it was prevalent to invite team leaders to evaluate team creativity (e.g., Shin and Zhou, 2007), future research should adopt an objective measurement for team creativity to improve the robustness of the results. Thirdly, we focused on the team level. As suggested, future research in the field of workplace demographic diversity should attempt to bridge the macro and micro theoretical domains (Joshi and Roh, 2009) with more concern for cross-level contextual variables and multilevel research. In addition, it would be more convictive to measure the particular shape of the educational level distribution for arguing that educational level is not a unitary construct as its effect on team creativity. Further, the follow-up studies are required to investigate other moderators and underlying mediators in the relationship between educational level diversity and team creativity.

## Conclusion

This study focuses on the separation and disparity form of educational level. Based on an integrative “status characteristics–information elaboration” theoretical framework, we propose that task and personnel variability are important contextual factors that moderate the effect of educational level diversity on team creativity. When teams were confronted with more variable tasks or fewer personnel changes, educational level diversity was more likely to facilitate team creativity.

## Data Availability Statement

The raw data supporting the conclusions of this article will be made available by the authors, without undue reservation.

## Ethics Statement

The studies involving human participants were reviewed and approved by the Research Ethics Board of the Academy of Neuroeconomics and Neuromanagement in Ningbo University. The patients/participants provided their written informed consent to participate in this study.

## Author Contributions

WG takes charge of the conduct of the study, including the conception of the work, the collection, analysis and interpretation of data, as well as drafting and revising the manuscript. CG contributed to data collection, interpretation, and the revision of the manuscript. DW made substantial contributions to the conception of the work and the revision of the manuscript. All authors contributed to the article and approved the submitted version.

## Conflict of Interest

The authors declare that the research was conducted in the absence of any commercial or financial relationships that could be construed as a potential conflict of interest.
